# There is a party in my head and no one is invited: Resting‐state electrocortical activity and solitude

**DOI:** 10.1111/jopy.12876

**Published:** 2023-08-14

**Authors:** Chengli Huang, James W. Butterworth, Anna J. Finley, Douglas J. Angus, Constantine Sedikides, Nicholas J. Kelley

**Affiliations:** ^1^ Centre for Research on Self and Identity, School of Psychology University of Southampton Southampton UK; ^2^ Institute on Aging University of Wisconsin‐Madison Madison Wisconsin USA; ^3^ School of Psychology Bond University Robina Queensland Australia

**Keywords:** approach–avoidance motivation, resting‐state EEG, self‐determination theory, solitude

## Abstract

**Objective:**

What are the motivational underpinnings of solitude? We know from self‐report studies that increases in solitude are associated with drops in approach motivation and rises in avoidance motivation, but only when solitude is experienced as non‐self‐determined (i.e., non‐autonomous). However, the extent to which individual differences in solitude relate to neurophysiological markers of approach–avoidance motivation derived from resting‐state electroencephalogram (EEG) is unknown. These markers are Frontal Alpha Asymmetry, beta suppression, and midline Posterior versus Frontal EEG Theta Activity.

**Method:**

We assessed the relation among individual differences in the reasons for solitude (i.e., preference for solitude, motivation for solitude), approach–avoidance motivation, and resting‐state EEG markers of approach–avoidance motivation (*N* = 115).

**Results:**

General preference for solitude was negatively related to approach motivation, observed in both self‐reported measures and EEG markers of approach motivation. Self‐determined solitude was positively related to both self‐reported approach motivation and avoidance motivation in the social domain (i.e., friendship). Non‐self‐determined solitude was positively associated with self‐reported avoidance motivation.

**Conclusion:**

This research was a preliminary attempt to address the neurophysiological underpinnings of solitude in the context of motivation.

## 1 INTRODUCTION

I find it wholesome to be alone the greater part of the time. To be in company, even with the best, is soon wearisome and dissipating. I love to be alone. I never found the companion that was so companionable as solitude. Henry David Thoreau, *Walden* (1854, p. 104)

In popular media and classic American literature, the experience of solitude is sometimes considered positive, as the song that inspired the title of our article, Tame Impala's *Solitude is Bliss*, attests: “There's a party in my head and no one is invited” (released in April 2010). However, solitude shares several characteristics with unpleasant, and often harmful, experiences such as loneliness or isolation. Solitude may also conduce to loneliness and isolation. The discrepancy between different faces of solitude may be closely tied to the motivations for seeking it out. We examine this possibility from a resting state electrocortical activity standpoint: To what extent is variation in the experience of solitude associated with variation in the electrocortical activity linked to motivation?

### 1.1 A brief history of solitude in psychology

In the early days of psychology, the topic of solitude gained eminence among humanists and psychoanalysts. In his pioneering book *Motivation and Personality*, humanistic psychologist Abraham Maslow (1954) listed solitude as one of the 15 most important characteristics of self‐actualized individuals. From the perspective of emotional development, psychoanalyst Donald Winnicott (1958) regarded solitude as the capacity to be alone and assumed solitude to be a key signal of an individual's emotional maturity. Similarly, psychoanalyst Anthony Storr (1988) posited that solitude could be as therapeutic as emotional support, promote insight, and facilitate contact with one's inner life. However, this early theorizing was unaccompanied by evidence.

Empirical research on solitude emerged in the 1990s when researchers began to develop definitions of it. Larson (1990) argued that solitude is not defined by physical separation from people, but by separation of communication and control, the severance of exchange of information and affect despite the presence of others (e.g., waiting on a bus, sitting in a coffee shop). Similarly, Burger (1995) defined solitude as the absence of social interaction, no matter in physical isolation or the presence of others. Long and Averill (2003) conceptualized solitude as a state of “being or living alone,” “seclusion,” and “solitariness,” but not necessarily loneliness. Therefore, solitude is an objective state of being alone, defined by the communicative severance of others, and does not have a specific valence connotation (Lay et al., 2019).

Rather than defining solitude per se, scholars have deconstructed it multidimensionally. For example, Burger (1995) postulated that solitude comprises the avoidance of social interaction as well as the appreciation of benefits from spending time alone. Larson and Lee (1996) differentiated between involuntary solitude and deliberately structured solitude. Larson (1997) later took volition into consideration, and further distinguished between misanthropic solitude which is a pure avoidance of social situations, and constructive solitude which is strategic retreating from social life. Later, Long et al. (2003) classified solitude into three dimensions: inner‐directed solitude (characterized by self‐discovery and internal peace), outer‐directed solitude (characterized by intimacy and spirituality), and loneliness (characterized by negative affect cooccurring with episodes of solitude). Finally, taking a developmental approach, Coplan and Armer (2007) drew theoretical and empirical distinctions among multiple forms of solitude: shyness, social disinterest, social avoidance, and reasons for young children's preference to play alone. In all, solitude remains a somewhat elusive concept. This assertion is further underscored by the multitude of motivations that people have for its pursuit.

Inspired by self‐determination theory (Deci & Ryan, 1985; Ryan & Deci, 2000), Nicol (2005) proposed that people engage in solitude to fulfill intrinsic motivations and extrinsic motivations, termed *self‐determined solitude* and *non‐self‐determined solitude*, respectively. For example, some are motivated to keep away from others in order to self‐reflect and contemplate personal issues or important decisions (Burger, 1995), whereas others are motivated to be alone because of social anxiety and social rejection (Ren et al., 2016). Following Nicol's breakthrough, researchers have re‐interpreted solitude from a motivational perspective (Borg & Willoughby, 2022; Chua & Koestner, 2008; Nguyen et al., 2018, 2019; Thomas & Azmitia, 2019; van Zyl et al., 2018; Yuan & Grühn, 2022). In line with these advances, we approach reasons for solitude from both a general level (assessing a general preference for solitude) and a specific level (distinguishing between self‐determined reasons and non‐self‐determined reasons for it).

### 1.2 Examining different solitude experiences in the context of approach–avoidance motivation

#### 1.2.1 The paradox of solitude

History and philosophy are littered with examples of the virtues of solitude. In the West, transcendental philosophers, as the opening quote from Thoreau indicates, emphasized the solitary self. In the East, Confucianism described Jun‐Zi (“君子”), a man of virtue, as achieving inner peace through solitude (“君子慎独”). Moreover, religious leaders (e.g., Buddha, Mohammed) and stoic philosophers (e.g., René Descartes, Immanuel Kant) spent a considerable amount of time in solitude and finished their masterpieces in a solitary life. Taken together, examples from history and philosophy suggest that solitude is a psychological venue for quiet reflection and can be a source of inspiration and creativity (Rubin et al., 2014).

Nonetheless, solitude's reputation remains mixed or negative. Humans have a fundamental need to form and maintain social relationships (Baumeister & Leary, 1995). When these needs are thwarted, loneliness can unfold (Leary, 2015) with negative ramifications for psychological and physical health (Cacioppo & Cacioppo, 2018). As such, the importance of social relationships and pernicious effects of loneliness have led researchers to cast other states of aloneness (i.e., solitude) either negatively or ambivalently (Larson, 1990; Lay et al., 2019; Pauly et al., 2017; Wang et al., 2013). Why have scholars formulated such divergent views on solitude? The motivation for solitude may influence how solitude is interpreted.

#### 1.2.2 Solitude and approach–avoidance motivation

The most rudimentary of motivational processes is approach–avoidance motivation: Even simple organisms, like the dark‐adapted earthworm, approach darkness by elongating and contracting their bodies to avoid the painful, aversive sunlight (Schneirla, 1959). As species up the evolutionary ladder become more complex so too does the manifestation of approach–avoidance motivation being focused more so on the approach to satisfaction and avoidance of threats (Schutter & Van Honk, 2005; Van Honk & Schutter, 2006). Accordingly, approach motivation exists in behaviors energized or directed toward desirable stimuli, whereas avoidance motivation exists in behaviors energized or directed by undesirable stimuli (Elliot, 1999, 2006; Elliot & Thrash, 2002). In addition, approach and avoidance are conceptualized as largely independent motivational tendencies (Asendorpf, 1990; Elliot, 1999), and evidence indicates that approach motivation and avoidance motivation are distinct (Ebner et al., 2006). Insofar as approach–avoidance motivation is a fundamental building block of human social behavior (Elliot et al., 2006), we consider the possibility that it also underlies the different experiences of solitude.

##### Solitude and approach motivation

Approach motivation is expressed in humans partially through interest and enjoyment of social activities (Elliot et al., 2006; Elliot & Friedman, 2007). Individuals motivated to be solitary tend to remove themselves from or engage in less social activities. Based on this logic, some theorists have suggested that solitude is associated with low approach motivation (Asendorpf, 1990; Coplan et al., 2015; Coplan & Armer, 2007). Consistent with this view, the frequency and enjoyment of solitary activities were negatively related to the desire for social contact (Leary et al., 2003), while. greater preference for solitude is associated with blunted approach motivation (Hassan et al., 2021). Therefore, we hypothesize that solitude, as a state of disconnection from communicating with others, is associated with low approach motivation whatever the reason for solitude.

Humans are social by nature, and social connection is a psychological need that can contribute to health and well‐being (Baumeister & Leary, 1995). However, too much of a good thing can be a bad thing. Excessive sociality is unassociated with and may be harmful to, health and well‐being (Santini et al., 2021; Stavrova & Ren, 2021). Indeed, like sociality, solitude appears to be a psychological need (Buchholz, 2000; Buchholz & Catton, 1999). For example, in a 2‐week long daily diary study, more than half of high school students reported that they needed solitude and regarded it as a priority in their lives (Freeman et al., 1986). Therefore, a balance between sociality and solitude appears beneficial to one's solitude experiences.

##### Solitude and avoidance motivation

Only a few studies have examined the association between reason for solitude and avoidance motivation. Some theorists argued that high avoidance motivation is not the reason why unsociable children are less involved with peers, as they might merely be more interested in playing with objects than people (Asendorpf, 1990). Also, some researchers reported that the preference for solitude is associated with elevated avoidance motivation (Hassan et al., 2021). However, the Preference for Solitude Scale used in the latter study contained items conceptually related to social avoidance (e.g., “I prefer spending Friday night alone rather than being with others”). Given that this scale is a general index of solitude, it is difficult to paint a clear picture between it and avoidance motivation. Nevertheless, such a picture is possible when one takes the specific motivations for solitude into consideration, (i.e., self‐determined solitude, non‐self‐determined solitude).

From the perspective of self‐determination theory, autonomy refers to being the perceived origin or source of one's behavior; non‐self‐determined motivated behaviors are less autonomous than self‐determined motivated ones (Ryan & Deci, 2002). Given that solitude can be driven by both self‐determined and non‐self‐determined reasons, the link between reason for solitude and avoidance motivation might depend on the degree to which solitude is autonomous. Moreover, people seek autonomy, as it is a fundamental psychological need (Ryan & Deci, 2002). Therefore, driven by self‐determined motivation, self‐determined solitude (e.g., being content with solitude) is associated with more volition and autonomy, and hence accompanied by low avoidance motivation. On the other hand, driven by non‐self‐determined motivation, non‐self‐determined solitude (e.g., being actively excluded by others) is associated with less volition and autonomy, and hence accompanied by high avoidance motivation.

Based on this logic, avoidance motivation colors the experience of solitude. Involuntary anxious solitude (i.e., passive anxious withdrawal) in the context of high exclusion is associated with persistent or exacerbated social avoidance (e.g., socially helpless behavior) over the course of a year (Gazelle & Rudolph, 2004). Additionally, when individuals autonomously spend time alone (low avoidance motivation), they report lower loneliness, higher psychological well‐being (Chua & Koestner, 2008; Nguyen et al., 2019), increased relaxation, and reduced stress (Nguyen et al., 2018). However, individuals engaging in solitude involuntarily (e.g., anxiety‐based avoidance from others—a high avoidance motivation) manifest more loneliness, anxiety, and depression (Thomas & Azmitia, 2019).

### 1.3 The measurement of solitude

There are two main approaches to measuring solitude. The first is self‐report questionnaires. For example, Burger (1995) developed the Preference for Solitude Scale to assess individual differences in solitude at a general level. Larson and Lee (1996) developed the Capacity to Be Alone Scale, which consists of a solitary coping subscale concerning the use of solitude to handle stress and a solitary comfort subscale concerning people's emotional comfort or discomfort in solitude. Long et al. (2003) proposed a multi‐dimensional solitude scale to assess inner‐directed solitude, outer‐directed solitude, and loneliness. Grounded in self‐determination theory, Nicol (2005) proposed the Motivation for Solitude Scale to measure the motivation for solitude, and later Thomas and Azmitia (2019) developed a short‐form, Motivation for Solitude Scale—Short‐Form (MSS‐SF). Galanaki et al. (2015) constructed the Children's Solitude Scale to assess individual differences in the use of voluntary solitude (self‐reflection, autonomy/privacy, activities, and concentration) during middle and late childhood. Recently, Palgi et al. (2021) focused on the positive aspects of volitional solitude proposing the Positive Solitude Scale. These questionnaires are commonly used and allow researchers both general and differentiated assessments of solitude.

Another way to assess solitude involves the Experience Sampling Method (Csikszentmihalyi & Larson, 1987). In relevant studies, researchers send signals to various devices (e.g., electronic pagers) carried by participants at randomized points throughout normal waking hours, and participants complete solitude‐related questionnaires or other ratings based on their feelings at the moment the signal is received (Larson, 1990; Matias et al., 2011; Thomas et al., 2021). Given that it can collect real‐time data in natural situations, this method ensures ecological validity unmatched by self‐report (Csikszentmihalyi & Larson, 1987). However, ecological validity comes at the cost of time‐consuming collection and complex data processing or analyses (Li & Zheng, 2008).

In the current study, like the scarce research linking the reasons for solitude to approach–avoidance motivation, we also use self‐report questionnaires. Specifically, we investigate the reasons for solitude with both a general measure (the Preference for Solitude Scale) and a specific measure that distinguishes between self‐determined reasons and non‐self‐determined reasons for solitude (Motivation for Solitude Scale). To circumvent the common method variance problem in assessing approach–avoidance motivation, introduced by relying exclusively, and serially on self‐report questionnaires (Biderman et al., 2011), we also measure electrophysiological markers of approach–avoidance motivation.

### 1.4 Electrophysiological markers of approach–avoidance motivation

For nearly a century, electroencephalography (EEG) has been the predominant *direct* measure of neural activity. EEG reflects the synchronous activity of populations of cortical neurons, and resting state EEG represents stable patterns in this activity when participants are unengaged in a task (Khanna et al., 2015). Major personality theories propose that personality is instantiated in the brain (Allport, 1937; Eysenck, 1967). That is, individual differences in neural activity may drive the stable patterns of emotion, cognition, and behavior that researchers call “personality.” However, work linking individual differences in personality to individual differences in neural activity is still in its infancy. Consistent with a personality neuroscience perspective, we examined the relation between individual differences in the reasons for solitude and individual differences in resting state EEG markers of approach–avoidance motivation as asymmetric frontal cortical activity, beta suppression, and Posterior versus Frontal EEG Theta Activity (PFTA).

#### 1.4.1 Asymmetric frontal cortical activity

One of the most frequently studied neural markers of approach–avoidance motivation is asymmetric frontal cortical activity, which refers to lateralized patterns of activity typically derived from EEG activity (Davidson, 1988). In particular, on alpha band power (8–12 Hz), greater relative left Frontal Alpha Asymmetry (FAA), especially over the prefrontal cortex, is associated with approach‐motivated traits (Coan & Allen, 2003; Harmon‐Jones & Allen, 1997, 1998), whereas greater relative right FAA is associated with avoidance‐motivated traits (Coan et al., 2001; Dawson et al., 1992). Similar results have been obtained in studies examining state‐like variation in approach–avoidance (Davidson et al., 1990; Harmon‐Jones, 2007; Harmon‐Jones et al., 2003, 2006; for a review, see Harmon‐Jones & Gable, 2018). Also, lateralized patterns of frontal Beta Power (BP; 13–30 Hz) might underlie approach–avoidance motivation (Schutter et al., 2008). In all, asymmetrical frontal cortical activity is a neural marker of approach–avoidance motivation.

#### 1.4.2 Beta suppression

One crucial aspect of approach–avoidance motivation is the direction of physical movement. Approach motivation entails physically moving toward a stimulus, whereas avoidance motivation entails moving away from a stimulus (Harmon‐Jones et al., 2013). Beta band activity (13–30 Hz) measured by EEG over the motor cortex is associated with approach motivation in terms of physical movement, which emerges in the context of both real and imagined motor movements (McFarland et al., 2000). Beta suppression^1^ (i.e., lower levels of beta activation) is associated with task‐induced state approach motivation (Gable et al., 2016) and behavioral reactions to approach‐oriented stimuli (Pluta et al., 2018; Wilhelm et al., 2021). Also, for individual differences in beta activation, lower levels of resting beta activity are linked to greater trait approach motivation (Threadgill & Gable, 2018). Furthermore, beta suppression over the motor cortex is related to another neural correlate of motivation (i.e., greater left FAA; Wendel et al., 2021). In summary, beta suppression over the motor cortex is a marker sensitive to motoric aspects of approach motivation.

#### 1.4.3 Posterior versus Frontal EEG Theta Activity

Midline PFTA, which constitutes a difference score between posterior (Pz) and frontal (Fz) midline theta activity, has also emerged as a promising neural marker of approach motivation (Wacker et al., 2006). Resting state PFTA and self‐reported approach‐related motivation are positively linked (Wacker et al., 2010; Walden et al., 2015), as are resting state PFTA and approach‐related traits (Chavanon et al., 2011; Wacker et al., 2006; Wacker & Gatt, 2010). To experimentally attenuate approach, one could reduce or prevent participants' ability to act (Kelley et al., 2013; Zinner et al., 2008). One study prevented participants from acting by exposing them to uncontrollable (vs. controllable) aversive noise blasts and found that PFTA decreased in response to the uncontrollable noise blasts (Reznik et al., 2017). Finally, both PFTA and approach motivation are linked to mesolimbic dopamine (Wacker et al., 2006, 2013; Wacker & Smillie, 2015). Insofar as mesolimbic dopamine underlies wanting (vs. linking or learning about) rewards (Robinson et al., 2005), PFTA may be driven more strongly by goal‐striving aspects of the Behavioral Activation System (BAS) analogous to Carver and White's (1994) drive subscale.

### 1.5 Overview

The approach–avoidance motivation system is a fundamental building block of human social behavior. Attempts to link this system to reasons for solitude have been limited and focused on self‐report measures. No published studies have examined the extent to which individual differences in reasons for solitude relate to three common neurophysiological markers of approach–avoidance motivation derived from resting‐state EEG: FAA, beta suppression, and PFTA. Although all of these neurophysiological markers reflect the activity of the approach–avoidance motivational system, they may represent different aspects of the system. FAA has its roots in the study of emotion (for a review, see Reznik & Allen, 2018) and may denote an affective component of approach–avoidance motivation. Beta suppression is robustly linked to real or imagined motor behavior and may represent a motoric aspect of approach motivation. Finally, PFTA may signify goal‐striving tendencies. By assessing simultaneously the relation between reasons for solitude and all three of these markers, we are equipped to ask: Are the reasons for solitude related to the affective, motoric, and/or goal‐striving aspects of the approach–avoidance motivational system? Our research, then, promises to clarify the mechanisms underlying the reasons for solitude and contribute to the emerging field of personality neuroscience.

#### 1.5.1 Hypotheses

##### 1.5.1.1 Hypothesis 1

Solitude (i.e., preference for solitude, self‐determined solitude, non‐self‐determined solitude) is negatively related to self‐reported approach motivation.

##### 1.5.1.2 Hypothesis 2

Solitude relates to avoidance motivation differently as a function of self‐determined motivation. Specifically, self‐determined solitude is negatively related to self‐reported avoidance motivation, whereas non‐self‐determined solitude is positively related to self‐reported avoidance motivation.

##### 1.5.1.3 Hypothesis 3

Solitude (i.e., preference for solitude, self‐determined solitude, non‐self‐determined solitude) is negatively associated with neurophysiological markers of approach motivation (i.e., relative left FAA, beta suppression, PFTA).

##### 1.5.1.4 Hypothesis 4

Solitude relates to a neural marker of avoidance motivation (i.e., relative right FAA^2^) differently as a function of self‐determined motivation. Self‐determined solitude is negatively associated with relative right FAA, whereas non‐self‐determined solitude is positively associated with relative right FAA.

## 2 METHOD

### 2.1 Ethical approval

The research protocol has been approved by the Ethics Committee of University of Southampton.

### 2.2 Participants

To determine the sample size for the association between reasons for solitude and three neural markers of approach motivation, we conducted an a priori power analysis using G*Power 3.1 (Faul et al., 2007). We aimed for 90% power assuming a two‐sided test and *α* = 0.05. For this effect size, a previous investigation indicated a moderately sized association between the reason for solitude (i.e., Preference for Solitude) and self‐report approach motivation (*r* = 28 to 0.35; Hassan et al., 2021, Study 1). We elected to recruit a sample sufficient to detect an intermediate effect (*r* = 0.30 ≈ *f* ^*2*^ = 0.0986) between the lower (*r* = 0.28) and higher (*r* = 0.35) effects observed by Hassan et al. (2021). With this effect size, we needed 112 right‐handed participants^3^ to detect bivariate associations with 90% power. To account for potential data loss or exclusions (e.g., unusable resting spectral power EEG data, greater than 50% of EEG data rejected, left‐handedness), we recruited 144 undergraduate students from University of Southampton for course credit. According to criteria of Stage 1 Registered Report, we excluded six participants due to bad voltage EEG signal in the data analyses, and 25 participants due to being mixed‐handed or left‐handed. Thus, we based the final analyses on data from 115 participants (94 men, 21 women). Their age ranged from 18 to 45 years (*M* = 19.77, *SD* = 3.46).

### 2.3 Procedure

Participants completed all measures and tasks in a soundproof laboratory room. At first, they completed an 8‐min resting state EEG data collection (4 mins with eyes open, 4 mins with eyes closed; Threadgill & Gable, 2018).^4^ During the resting‐state session, they were required to keep as still as possible, and they were instructed to visually fixate on a cross presented on the computer screen when opening their eyes. The resting‐state session itself is in a neutral environment, and not meant to manipulate state solitude. Then, participants undertook a self‐reference task^5^ (as part of a different project), in which they judged whether or not a list of traits described themselves (D'Argembeau et al., 2005). Finally, participants filled out the solitude‐related questionnaires (described below). Debriefing concluded the experimental session.

### 2.4 Materials

#### 2.4.1 Preference for Solitude Scale

We slightly adapted the 16‐item Preference for Solitude Scale (Ren et al., 2021). Participants indicated the extent to which each item (e.g., “I need time alone each day”, “I do not understand people who choose to be alone” [reverse coded]) applied to them (1 = *not at all*, 7 = *very much*; *α* = 0.89) instead of being presented with binary choices as in the original scale (Burger, 1995). Higher values reflected stronger preference for solitude.

#### 2.4.2 Motivation for Solitude Scale‐Short Form

We used the 14‐item Motivation for Solitude Scale‐Short Form (MSS‐SF; Thomas & Azmitia, 2019) to measure specific indices of solitude (i.e., self‐determined, non‐self‐determined solitude). The 8‐item Self‐Determined Solitude (SDS) subscale assesses the importance of being alone for intrinsic reasons (e.g., “I can engage in activities that really interest me”; *α* = 0.81). The 6‐item Non‐Self‐Determined Solitude (NSDS) subscale assesses the importance of being alone for extrinsic reasons (e.g., “I feel uncomfortable when I'm with others”; *α* = 0.89). Participants indicated the importance of each item (1 = *not at all important*, 7 = *extremely important*). We averaged responses to the SDS and NSDS, with higher values representing more self‐determined solitude and more non‐self‐determined solitude, respectively.

#### 2.4.3 Domain‐general and domain‐specific measures of approach–avoidance motivation

##### BIS/BAS

We assessed domain‐general approach–avoidance motivation with the 20‐item Behavioral Inhibition System and Behavioral Approach System (BIS/BAS) Scale (Carver & White, 1994). The 7‐item BIS gauges reactions to aversive stimuli (e.g., “Criticism or scolding hurts me quite a bit”). The 13‐item BAS comprises three subscales. The 5‐item Reward Responsiveness subscale measures responses to real (e.g., “When I see an opportunity for something I like I get excited right away”) and imagined (e.g., “It would excite me to win a contest”) rewards. The 4‐item Drive subscale measures goal‐striving tendencies (e.g., “I go out of my way to get things I want”). Finally, the 4‐item Fun‐seeking subscale measures sensation seeking (“I crave excitement and new sensations”) and impulsivity (“I often act on the spur of the moment”). Although the BAS was originally conceived as a multi‐dimensional scale, we used a composite score for two reasons. First, a general factor or BAS total score is meaningful and interpretable above and beyond the three subscales (Kelley et al., 2019). Second, our hypotheses were not specific to a particular BAS facet. Participants indicated their level of agreement with each BIS/BAS item (1 = *extremely true for me*, 7 = *extremely false for me*). We averaged responses to BIS/BAS, such that higher values were indicative of stronger approach/avoidance motivation. Alphas for BIS and BAS were 0.79 and 0.85, respectively. All alphas for the BAS subscales were acceptable (0.72–0.82).

##### Social approach–avoidance motivation

We assessed domain‐specific approach–avoidance motivation with the 8‐item friendship‐approach and friendship‐avoidance goals scale (Elliot et al., 2006). The 4‐item Social Approach Motivation (SAPM) subscale gauges motivation to strengthen or enhance friendships (e.g., “I am trying to deepen my relationships with my friends”). The 4‐item Social Avoidance Motivation (SAIM) subscale gauges motivation to avoid harming friendships (e.g., “I am trying to avoid disagreements or conflicts with my friends”). Participants indicated their level of agreement with each item (1 = *extremely true for me*, 7 = *extremely false for me*), with higher values reflecting stronger social approach/avoidance motivation. We averaged the friendship‐approach and friendship‐avoidance goals scale to create indices of the SAPM (*α* = 0.84) and SAIM (*α* = 0.86) subscales.

### 2.5 Data collection and analyses

#### 2.5.1 EEG collection and processing

We collected EEG data continuously from 64 scalp sites using Ag/AgCl electrodes mounted in an elastic cap (Neuroscan, NC), with an online reference to the left mastoid and off‐line algebraic re‐reference to the average of left and right mastoids. We mounted a ground electrode midway between AFz and Fz. We recorded the vertical electrooculogram (VEOG) and horizontal electrooculogram (HEOG) from two pairs of electrodes, with one placed above and below the left eye, and another placed 10 mm from the outer canthi of each eye. The electrode cap is based on the 10–20 system. We kept electrode impedances below 5 kΩ. We amplified and sampled signals at 1000 Hz with an online bandpass filter from 0.1 to 100 Hz.

In off‐line processing, we combined data from open and closed eyes together, and initially pre‐processed all the EEG data using EEGLAB, an open‐source toolbox running in the MATLAB environment (Delorme & Makeig, 2004). We band‐passed filter continuous EEG data (low pass: 0.1 Hz, high pass: 40 Hz, 50 Hz notch). We segmented the continuous combined eyes open and eyes closed data into 2000 ms windows overlapping by 50%. We replaced bad channels using a spherical spline interpolation (SSI; Perrin et al., 1989). We corrected segments contaminated by blink, eye movement, and other artifacts using an Independent Component Analysis (ICA) algorithm (Delorme & Makeig, 2004) and ADJUST, a completely automatic algorithm for artifact identification and removal in EEG data. ADJUST has a similar performance to manual rejection by expert analysts (agreement on 95.2% of the data variance; Mognon et al., 2011). We excluded bad segments if there was a voltage deviation on any channel of ±70 μV. We excluded participants with more than 50% of the total number of segments rejected from analyses in a listwise fashion. Finally, we applied a fast Fourier transformed (FFT) to the processed EEG data to calculate spectral power for different frequency bands: theta (4–7 Hz), alpha (7–13 Hz), and beta (13–30 Hz).

#### 2.5.2 Resting EEG neurophysiological markers

We calculated FAA by subtracting log normalized left hemisphere values from log normalized right hemisphere values for F4/F3 [natural log of power at F4 minus natural log of power at F3], F6/F5 [natural log of power at F6 minus natural log of power at F5], and F8/F7 [natural log of power at F8 minus natural log of power at F7]^6^ (Barnhofer et al., 2007; Davidson, 1988). We averaged power spectra for the alpha band among frontal sites F4‐F3, F6‐F5, and F8‐F7.^7^ Given that cortical power is inversely related to cortical activity (Davidson, 1988), higher scores of this metric indicate relative right hemisphere cortical power, which corresponds to larger cortical resource allocation in the left hemisphere (i.e., relative left FAA; Briesemeister et al., 2013). Given that FAA is a continuous index, lower scores reflect relative right FAA and higher scores reflect relative left FAA. We used this index to test Hypotheses 3 and 4.

We transformed resting BP value over motor cortices (C1, C2, C3, C4, C5, C6, CP1, CP2, CP3, CP4, CP5, CP6; Gable et al., 2016; Wendel et al., 2021) using natural logarithms to produce normal distributions (Davidson et al., 2000). We averaged power spectra for the beta band across the regions of the head at sites over the motor cortices (Wendel et al., 2021).^8^ Lower beta activity indicates greater beta suppression. We calculated theta PFTA by subtracting log normalized parietal cortical values from log normalized frontal cortical values [natural log of power at Fz minus natural log of power at Pz] (Wacker et al., 2006).

### 2.6 Statistical analyses

We carried out statistical analyses in SPSS 24.0 software for Windows (version 10). Given that the data were not normally distributed after logarithmic transformation, we conducted Spearman correlational analyses^9^ (*α* = 0.05, two‐tailed) to examine the correlations between self‐reported questionnaires (i.e., PSS, SDS, NSDS, BAS, BIS, SAPM, and SAIM) and resting‐state EEG neurophysiological markers (i.e., FAA, beta suppression, and PFTA).

#### 2.6.1 Multiple comparisons

Across all four hypotheses, there were 19 focal tests.^10^ Six correlations contributed to our test of Hypothesis 1. Four correlations contributed to our test of Hypothesis 2. Nine correlations contributed to our test of Hypotheses 3 and 4. In addition to evaluating the significant of the focal tests above, we corrected for multiple comparisons using the Benjamini–Hochberg procedure (Benjamini & Hochberg, 1995). This approach is a statistically more powerful way to deal with false discovery rate in multiple comparisons as compared to a Bonferroni correction (Williams et al., 1999). The Benjamini–Hochberg procedure sequentially ranks each *p*‐value and compares them to a Benjamini–Hochberg critical value. The critical value is computed as a function of the rank (*k*), the number of tests (*n*), and the alpha level (*α* = 0.05), i.e., (*k*/*n*) * *α*. Using this procedure, we offer an additional way to interpret the significance of our findings, if their *p*‐values are less than the corresponding Benjamini–Hochberg critical value. Finally, we conducted equivalence tests (Lakens et al., 2018), so we could interpret null results against the smallest effect size of interest. We selected *r* = 0.10 as the smallest effect size of interest, as it is considered a small effect size that is potentially consequential (Funder & Ozer, 2019) and a previous study with the same sample size as ours would have less than 33% power to detect that effect (Simonsohn, 2015).

## 3 RESULTS

We display in Table 1 means and standard deviations for self‐reported measures as well as mean and standard deviations for resting‐state EEG neurophysiological markers.

**TABLE 1:** Mean and standard deviations for self‐reported measures and resting‐state EEG neurophysiological markers.

	*M*	*SD*
*Self‐reported measures*		
Preference for Solitude Scale	3.922	1.019
Self‐Determined Solitude Subscale	4.655	1.061
Non‐Self‐Determined Solitude Subscale	2.843	1.323
Behavioral Approach System	5.055	0.949
Behavioral Inhibition System	5.398	1.234
Social Approach Motivation	5.861	0.998
Social Avoidance Motivation	5.798	1.084
*EEG markers*		
Frontal Alpha Asymmetry	−0.003	0.012
Beta Power	3.933	0.043
Posterior versus Frontal Theta Activity	0.036	0.017

We display in Table 2 the results of Spearman's correlation between solitude and approach–avoidance motivation (i.e., self‐reported measures, resting‐state EEG neurophysiological markers). In regard to Hypothesis 1 (i.e., solitude and self‐reported approach motivation), PSS was negatively related to approach motivation, as measured by the BAS (*r* = −0.200, *p* = 0.031), whereas SDS was positively related to approach motivation in the social domain, as measured by SAPM (*r* = −0.294, *p* = 0.001). However, NSDS did not exhibit a significant association with approach motivation (*p*s >0.05). In regard to Hypothesis 2 (i.e., solitude and self‐reported avoidance motivation), NSDS exhibited a positive association with avoidance motivation, as measured by BIS (*r* = −0.200, *p* = 0.031). However, SDS was also positively related to avoidance motivation in the social domain, as measured by SAIM (*r* = 0.196, *p* = 0.036).

**TABLE 2:** Spearman's correlations between solitude and approach–avoidance motivation (self‐reported measures, resting‐state EEG neurophysiological markers) across all participants.

	PSS	SDS	NSDS
*Self‐reported measures*			
BAS	−0.201*	0.031	−0.093
BIS	−0.076	−0.007	0.198*
SAPM	−0.067	0.294 **	0.021
SAIM	−0.040	0.196*	−0.024
*EEG markers*			
FAA	−0.180^†^	−0.104	0.015
BP	−0.221*	0.065	0.029
PFTA	0.023	−0.055	0.071

Abbreviations: BAS, Behavioral Approach System; BIS, Behavioral Inhibition System; BP, Beta Power; FAA, Frontal Alpha Asymmetry; NSDS, Non‐Self‐Determined Solitude Subscale; PFTA, Posterior versus Frontal EEG Theta Activity; PSS, Preference for Solitude Scale; SAIM, Social Avoidance Motivation; SAPM, Social Approach Motivation; SDS, Self‐Determined Solitude Subscale.

** *p* < 0.01

* *p* < 0.05

^†^ *p* < 0.10.

In regard to Hypotheses 3 and 4 (i.e., the neuroscientific aspect of solitude), we obtained significant negative correlations between PSS and resting‐state EEG neurophysiological markers, specifically FAA (*r* = −0.180, *p* = 0.054) and BP (*r* = −0.221, *p* = 0.018). As a reminder, higher FAA scores reflect relative left FAA, whereas lower FAA scores reflect relative right FAA. The trending negative correlations between PSS and FAA indicate that PSS was weakly negatively related to the relative left FAA (i.e., the EEG marker of approach motivation), and weakly positively related to the relative right FAA (i.e., the EEG marker of avoidance motivation). In terms of BP, given that a lower level of beta activation reflects larger beta suppression, the negative correlation between PSS and BP indicates a positive relation between PSS and beta suppression.

After Benjamini–Hochberg corrections, the positive relation between SDS and SAPM was still significant. See Table 3 for corrected *p*‐values (i.e., Benjamini–Hochberg critical values) for each correlation. See Appendix for supplementary analysis on correlation between self‐reported approach–avoidance motivation measures and motivation‐related EEG neurophysiological markers.

**TABLE 3:** Benjamini–Hochberg critical values for each correlation.

Test	*Rho*	Original *p*	*p*‐value rank (k)	B–H critical value	Significance
SDS × SAPM	0.294	0.001	1	0.003	**Significant**
PSS × BP	−0.221	0.018	2	0.005	Not significant
PSS × BAS	−0.200	0.031	3	0.008	Not significant
NSDS × BIS	0.198	0.034	4	0.011	Not significant
SDS × SAIM	0.196	0.036	5	0.013	Not significant
PSS × FAA	−0.180	0.054	6	0.016	Not significant
SDS × FAA	−0.104	0.270	7	0.018	Not significant
NSDS × BAS	−0.093	0.321	8	0.021	Not significant
NSDS × PFTA	0.071	0.453	9	0.024	Not significant
PSS × SAPM	−0.067	0.479	10	0.026	Not significant
SDS × BP	0.065	0.488	11	0.029	Not significant
SDS × PFTA	−0.055	0.559	12	0.032	Not significant
SDS × BAS	0.031	0.74	13	0.034	Not significant
NSDS × BP	0.029	0.76	14	0.037	Not significant
NSDS × SAIM	−0.024	0.798	15	0.039	Not significant
PSS × PFTA	0.023	0.804	16	0.042	Not significant
NSDS × SAPM	0.021	0.821	17	0.045	Not significant
NSDS × FAA	0.015	0.871	18	0.047	Not significant
SDS × BIS	−0.007	0.941	19	0.050	Not significant

Abbreviations: BAS, Behavioral Approach System; BIS, Behavioral Inhibition System; BP, Beta Power; FAA, Frontal Alpha Asymmetry; NSDS, Non‐Self‐Determined Solitude Subscale; PFTA, Posterior versus Frontal EEG Theta Activity; PSS, Preference for Solitude Scale; SAIM, Social Avoidance Motivation; SAPM, Social Approach Motivation; SDS, Self‐Determined Solitude Subscale.

**FIGURE 1:**
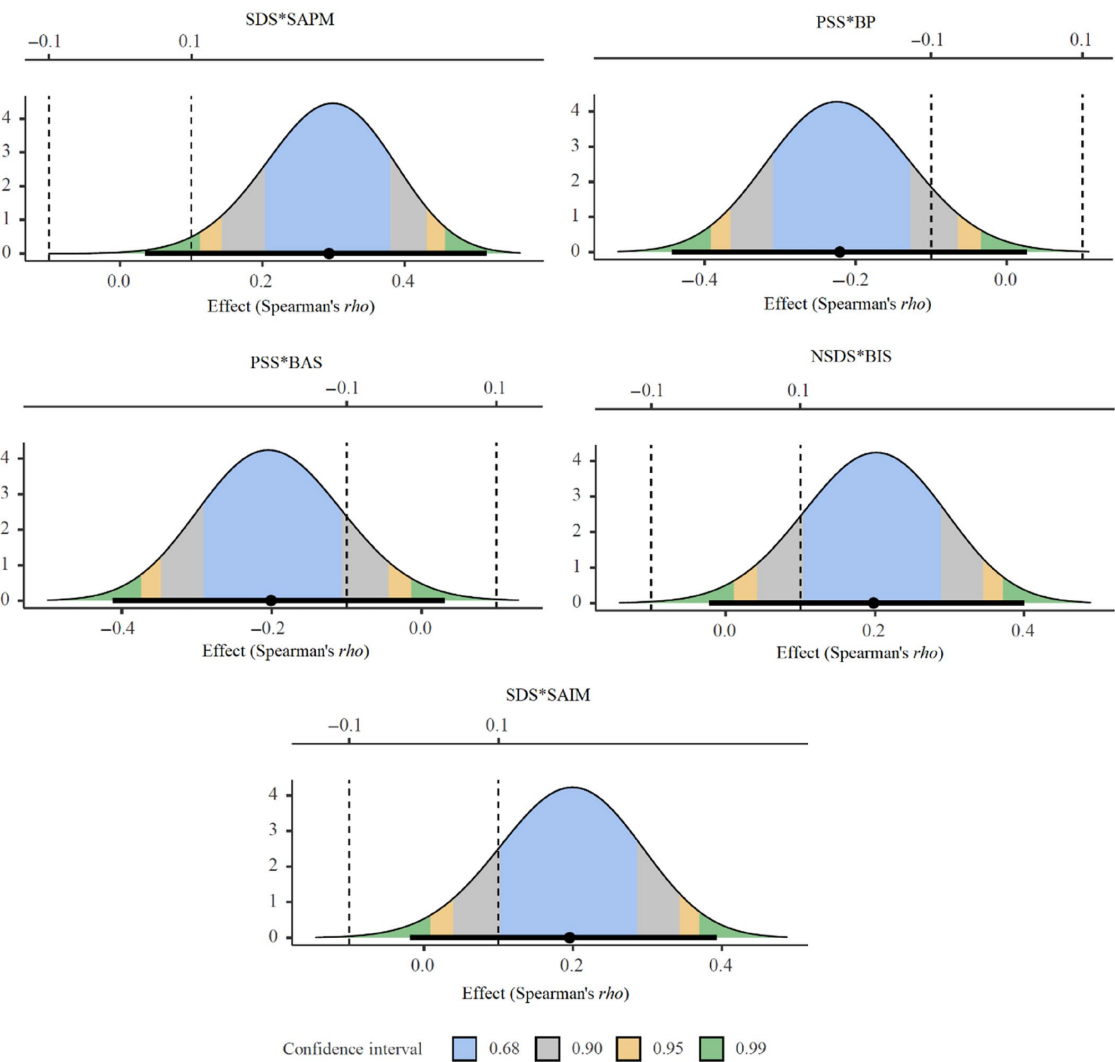
Equivalence tests for Spearman's correlations. In the plots, the thick horizontal lines indicated the confidence intervals from the two one‐sided tests (TOST) procedure, and the range of confidence intervals depends on the corrected *p*‐values (i.e., B‐H critical values). Take SDS × SPAM plot, for example, the confidence interval was 99.4% since the B–H critical value was 0.003. The dashed vertical lines indicated the equivalence bounds.

We present in Table 4 and Figure 1 the equivalence tests of the Spearman's correlations between solitude and approach–avoidance motivation following the Benjamini–Hochberg corrections. (In Figure 1, we only present the equivalence tests of significant Spearman's correlations before the Benjamini–Hochberg corrections. For the equivalence tests of null significant correlations after corrections, see Appendix). None of the equivalence tests was significant, *p*s >0.05, suggesting we cannot reject the null hypotheses that the true Spearman's correlations were at least as extreme as 0.10.

**TABLE 4:** Equivalence tests for Spearman's correlations.

Test	*Rho*	Confidence interval		Sig. results	TOST results
		Lower	Upper		
SDS × SAPM	0.294	0.036	0.515	True	False
PSS × BP	−0.221	−0.442	0.026	False	False
PSS × BAS	−0.200	−0.412	0.031	False	False
NSDS × BIS	0.198	−0.022	0.400	False	False
SDS × SAIM	0.196	−0.018	0.393	False	False
PSS × FAA	−0.180	−0.377	0.020	False	False
SDS × FAA	−0.104	−0.304	0.094	False	False
NSDS × BAS	−0.093	−0.283	0.104	False	False
NSDS × PFTA	0.071	−0.121	0.257	False	False
PSS × SAPM	−0.067	−0.250	0.122	False	False
SDS × BP	0.065	−0.118	0.245	False	False
SDS × PFTA	−0.055	−0.231	0.124	False	False
SDS × BAS	0.031	−0.145	0.206	False	False
NSDS × BP	0.029	−0.144	0.200	False	False
NSDS × SAIM	−0.024	−0.193	0.146	False	False
PSS × PFTA	0.023	−0.144	0.189	False	False
NSDS × SAPM	0.021	−0.143	0.184	False	False
NSDS × FAA	0.015	−0.145	0.178	False	False
SDS × BIS	−0.007	−0.165	0.152	False	False

Abbreviations: BAS, Behavioral Approach System; BIS, Behavioral Inhibition System; BP, Beta Power; FAA, Frontal Alpha Asymmetry; NSDS, Non‐Self‐Determined Solitude Subscale; PFTA, Posterior versus Frontal EEG Theta Activity; PSS, Preference for Solitude Scale; SAIM, Social Avoidance Motivation; SAPM, Social Approach Motivation; SDS, Self‐Determined Solitude Subscale.

## 4 DISCUSSION

An abundance of research has examined the structure of solitude, breaking it down into several constructs. Apart from estimating solitude at a general level, we set out to address its motivational correlates. We used motivation‐related EEG markers to provide unique insights into the nuance of solitude. To our knowledge, no previous research has explored the neurophysiological markers of approach–avoidance motivation in relation to solitude, despite some studies having linked solitude and related constructs (e.g., shyness, social disinterest, social withdrawal) to approach–avoidance motivation (Coplan et al., 2013; Coplan & Armer, 2007; Hassan et al., 2021). We found that solitude was positively related to avoidance motivation, and the association between solitude and approach motivation differed by self‐determination. We also obtained initial support for our hypothesis that general preference for solitude was related to different neurophysiological markers of approach–avoidance motivation both in the emotional and motoric aspects. Specifically, general preference for solitude was associated with reduced relative left FAA (i.e., the EEG marker of approach motivation) and increased relative right FAA (i.e., the EEG marker of avoidance motivation). Furthermore, general preference for solitude was associated with increased beta suppression. In all, general preference for solitude related differently to emotive (FAA) than motoric (beta suppression) aspects of approach motivation. However, these findings were no longer significant after correcting for multiple comparisons. Thus, we urge caution in interpreting them and a need for replicating them in larger samples.

### 4.1 Solitude and motivation

#### 4.1.1 Solitude and approach motivation

Our hypothesis about solitude and approach motivation was partly supported. Specifically, we only found a negative relation between general preference for solitude and BAS, a pattern consistent with prior results (Hassan et al., 2021). However, we did not find a negative relation between all three types of solitude (i.e., PSS, SDS, NSDS) and SAPM—approach to motivation in the social domain. Humans are social animals, and belongingness is considered a psychological need (Baumeister & Leary, 1995). To some extent, having a high preference for solitude do not necessarily imply a dislike for socializing. Compared to others, individuals characterized by higher preference for solitude are more likely to choose spending time by themselves rather than with others when both options are available (Burger, 1995). SAPM, indexed as the pursuit of friendship goals, is a common activity in the lives of young people (e.g., university students), who are eager to develop friendships, close relationships, or relationships in general. Thus, the relation between solitude and SAPM may not necessarily be negative. Indeed, this relation between SDS and SAPM was positive, an issue to which we return.

#### 4.1.2 Solitude and avoidance motivation

Our hypothesis about solitude and avoidance motivation was also partly supported. Specifically, we only found a positive association between NSDS and BIS. Self‐determination theory highlights the relevance of motivational processes (Deci & Ryan, 2000). NSDS implies passively being alienated from others due to external or internal pressures (e.g., peer rejection, social anxiety; Chua & Koestner, 2008; Thomas & Azmitia, 2019; van Zyl et al., 2018). Hence, involuntarily solitudinous individuals will be high on avoidance motivation, exhibiting strong reactions to aversive stimuli. However, we also unexpectedly observed a positive relation between SDS and SAIM (avoidance motivation in the social domain).

#### 4.1.3 SDS and motivation in the social domain

SDS was positively related to both SAPM and SAIM. Friendship represented approach and avoidance motivation in the social domain. Friendships are a developmental necessity throughout the life course (Hartup & Stevens, 1999). Especially in adolescence and emerging adulthood, forming and maintaining friendships is a key developmental task (Güroğlu, 2022) providing social scaffolding where social motives (e.g., engagement, intimacy, attachment, emotional support) can be met comfortably (Elliot et al., 2006). However, as a need parallel to friendship in development across the life span, the increasingly autonomous and independent self is relatively neglected (Nicol, 2005). Solitude is a state in which the dominant relationship is with the self (Weinstein et al., 2022). As an autonomous and voluntary solitude, SDS offers a constructive time for adolescents and emerging adults to engage in intrinsic motivations such as self‐reflection, identity formation, and creativity (Andre, 1991; Borg & Willoughby, 2022; Long & Averill, 2003; Thomas & Azmitia, 2019). Therefore, one explanation for the positive relation between SDS and both SAPM and SAIM is that people who enjoy self‐determined solitude have an affinity with social time (i.e., approach friendship) and solitary time (i.e., avoidance friendship), maintaining a balance between social connection and spend alone.

### 4.2 Solitude and motivation‐related EEG markers

The hypothesis about solitude and neural markers of approach motivation was partly supported. Specifically, we observed a trending negative relation between general preference for solitude and FAA. Greater left lateralized frontal cortical activity reflects the approach motivational system, whereas greater right lateralized frontal cortical activity reflects the avoidance motivational system (Coan & Allen, 2003; Jesulola et al., 2015). Both positive (Harmon‐Jones et al., 2008, 2011) and negative (Kelley et al., 2015; Li et al., 2016) approach‐motivated emotions are linked with greater relative left frontal asymmetry. Although greater relative left frontal asymmetry is associated with stronger approach‐motivated emotions, it is also associated with successful emotion regulation (Choi et al., 2016; Kim et al., 2012; Papousek et al., 2017). In our study, higher levels of solitude were associated with less relative left frontal asymmetry. Is this because highly solitudinous participants experience these strong approach‐motivated emotions less frequently or are they habitually worse at emotion regulation? To address this question, experimental approaches along with temporally and spatially precise neuroimaging techniques are needed.

The result of another neural marker of approach motivation (i.e., beta suppression) and solitude contradicted the hypothesis. Beta suppression (i.e., lower levels of beta activation) over the motor cortex is associated with a motoric aspect of approach motivation—no matter if it is the task‐induced state (Gable et al., 2016; Pluta et al., 2018; Wilhelm et al., 2021) or resting‐state trait (Threadgill & Gable, 2018)—namely, real or imagined motor behavior (McFarland et al., 2000; Pfurtscheller et al., 2005). However, we observed a negative relation between solitude and BP. Given that lower beta activity indicates greater beta suppression, general preference for solitude was associated with approach motivation pertaining to physical movement in the current study. This finding, although unexpected, is consistent with the idea that solitudinous people have a rich inner life replete of self‐reflection and creativity (Coplan et al., 2015; Long et al., 2003; Thomas & Azmitia, 2019). This mentalizing may be driven be replaying past or imaging future interactions or behaviors. This notion is consistent with studies showing greater beta suppression in the context of imagined movement. Studies are needed that: (1) directly and conceptually replicate associations between beta suppression and solitude, or in the context of relevant tasks in relation to solitude; (2) elucidate the mechanisms underlying these associations; and (3) examine whether beta suppression may explain links between solitude and creativity.

The solitude preference was differentially related to affective (i.e., negatively) and motoric (i.e., positively) components of motivation. One explanation for this contradictory outcome is that sociality, as a psychological need crucial for human survival and reproduction (Baumeister & Leary, 1995; Sedikides et al., 2006), is embedded within the human genetic framework. Consequently, individuals who exhibit a preference for solitude demonstrate a paradoxical readiness to engage in social interactions (motorically), driven by this need, despite displaying diminished approach motivation toward social activities (affectively). This intriguing finding contributes insight into the varying dynamics characterizing the association between solitude and different components of motivation.

We did not observe a significant association between solitude and PFTA. PFTA is linked to mesolimbic dopamine (Wacker et al., 2013; Wacker & Smillie, 2015), which underlies wanting (vs. linking or learning about) rewards (Robinson et al., 2005). As such, PFTA might be driven by goal‐striving aspects of the BAS. Thus, the null association between PFTA and solitude suggests that solitude might be unrelated to the goal‐striving aspects of the BAS. Experimental work, wherein goal striving is manipulated, could test this hypothesis.

### 4.3 Limitations

Results on the neurophysiological underpinnings of solitude with respect to approach–avoidance motivation were suggestive, but inconclusive. Although we considered the nuanced nature of solitude in terms of self‐determination, we were unable to provide robust evidence regarding the motivation orientation of solitude. We arrived at the sample size based on a moderately sized association between the PSS and BAS (*r* = 0.28 to 0.35; Hassan et al., 2021, Study 1), but the effects we observed were slightly smaller. It is possible that our study was underpowered, and a larger sample size would yield more definitive results. Additionally, whereas the Benjamini–Hochberg procedure is less conservative than traditional correction methods (e.g., Bonferroni correction; Benjamini & Hochberg, 1995; Benjamini & Yekutieli, 2001), it remains conservative when applied to discrete *p*‐values or mid and large *p*‐values (Bogdan et al., 2008; Chen & Sarkar, 2020). Consequently, a larger sample and a more sensitive, less conservative correction method might contribute to a comprehensive and dependable understanding of the relation between solitude and motivation.

Along with why one spends time alone, it important to understand how often one does so (Borg & Willoughby, 2022). We neglected the latter issue, although the balance between social and solitudinous time has psychological consequences (Coplan et al., 2018, 2019; Weinstein et al., 2022, 2023). For example, people who spend a moderate amount of time alone report higher well‐being than those who spent either low or high amounts of time alone (Csikszentmihalyi & Larson, 1987; Larson, 1997). Research will do well to address the interplay between motivation for solitude and frequency of its engagement.

Another limitation concerns FAA. The FAA analyzed for Hypothesis 4 (i.e., relative right FAA) was the same as for Hypothesis 3 (i.e., relative left FAA). Therefore, FAA metrics do not separate out the absolute levels of approach or avoidance motivation, but instead reflect the relative levels of approach compared to avoidance. The ability to discern and precisely quantify the extent of motivation is compromised, particularly in relation to avoidance motivation. The two types of motivation are distinct (Ebner et al., 2006), despite often being intertwined in daily life, adding a reason to address the said limitation. Also, future work should consider other avoidance motivation‐related neural markers, such as descending inhibition (negative correlations from the alpha to the delta system; Knyazev & Slobodskaya, 2003), to enrich empirical evidence for the relation between solitude and avoidance motivation.

Finally, resting‐state neural activity is not a unitary psychological experience. Rather it represents a diverse array of cognitive, emotive, perceptual, and motor processes. This diverse array of processes may vary as a function of solitude. To address this issue, future studies can employ retrospective interoceptive methods adaptive for neuroscientific research (Gonzalez‐Castillo et al., 2021) after resting‐state EEG. These techniques, well‐suited to detect trait‐like differences, will allow researchers to detect different psychological experiences during the resting‐state EEG. Are highly solitudinous participants engaging in creative thought, reliving past experiences, or imaging the future to a greater degree that those participants low in solitude? By answering such questions, research will clarify the mechanisms linking resting‐state EEG to solitude.

## 5 CONCLUSION

We provided preliminary evidence regarding solitude and neurophysiological markers of approach–avoidance motivation. The results revealed a mixed relation between solitude and motivation. General preference for solitude was negatively related to approach motivation, which we observed in both self‐reports and the biology marker of emotional approach motivation (i.e., FAA). In addition, general preference for solitude was positively related to approach motivation pertaining to physical movement. The SDS was a mixture of approach motivation and avoidance motivation in the social domain (i.e., friendship). The NSDS was primarily associated with avoidance motivation. We speculatively proposed a link between solitude and motivation, and the data partly supported this proposal. Future research may test more comprehensively the association between solitude and motivation.

## AUTHOR CONTRIBUTIONS

**Chengli Huang:** Conceptualization, Investigation, Data Curation, Formal Analysis, Visualization, Writing – Original Draft. **James W. Butterworth:** Conceptualization, Investigation, Writing – Review & Editing. **Anna J. Finley:** Conceptualization, Writing – Review & Editing. **Douglas J. Angus:** Conceptualization, Investigation, Writing – Review & Editing. **Constantine Sedikides:** Conceptualization, Supervision, Writing – Review & Editing. **Nicholas J. Kelley:** Conceptualization, Supervision, Writing – Review & Editing.

## CONFLICT OF INTEREST STATEMENT

The authors report no conflict of interest.

## Supporting information


Appendix S1

## Data Availability

All study data, analytic code, and study materials (e.g., questionnaires) are available at the Open Science Framework (OSF): https://osf.io/n8cg7/?view_only=918a58d1a0894d719ac82614675e361d.

## Endnotes

^1^
Some researchers have conceptualized beta suppression as task‐related reductions in beta activity in motor (vs. non‐motor) conditions (Gable et al., 2016; Pluta et al., 2018; Wilhelm et al., 2021). Others have conceptualized it as reductions in resting or tonic beta levels (Threadgill & Gable, 2018). We follow the latter conceptualization.

^2^
Relative left/right FAA are two parts of the same metric (i.e., FAA), not two different measures. Given that FAA is a continuous index, lower scores reflect relative right FAA and higher scores reflect relative left FAA. We used FAA to test Hypotheses 3 and 4.

^3^
Due to lateralization of emotive and motor processes studies of frontal asymmetry, beta suppression and PFTA are constrained to right‐handed participants.

^4^
It is conventional to record resting‐state EEG with both eyes open and eyes closed, because the power of EEG rhythms is different when people open or close their eyes, suggesting varying levels of arousal (Barry et al., 2007; Barry & De Blasio, 2017; Kan et al., 2017; Knyazev et al., 2002; Li, 2010).

^5^
Although some researchers posit that solitude is associated with self‐related topics, such as self‐reflection (Burger, 1995), self‐realization (Stern & Wałejko, 2020), and self‐regulation (Nguyen et al., 2018), there is no evidence that self‐reference/self‐evaluation directly affect solitude and motivation. Also, there was an approximately 30‐mintue physical cleaning session (i.e., participants cleaned up their hair) before the questionnaire administration. Therefore, we did not expect for the self‐reference task to impact the questionnaire responses.

^6^
In Stage 1 of the registered report, we only included F4/F3 and F8/F7 in the analysis. In Stage 2, we also involved F6/F5.

^7^
In Stage 1 of the registered report, we mentioned that we would calculate FAA by logging normalized hemisphere values, but we did not specify whether we would analyze FAA separately for each pair of sites or average them. In Stage 2, we averaged all pairs of sites.

^8^
In Stage 1 of the registered report, we mentioned that we would calculate BP value by logging normalized hemisphere values, but did not explicate whether we would analyze FAA separately for each site or average them. In Stage 2, we averaged across all sites.

^9^
In Stage 1 of the registered report, we wrote: “We will use correlational analysis (α = 0.05, two‐tailed) to evaluate the bivariate associations between self‐report measures of reasons for solitude and neural markers of approach motivation.” In Stage 2, we added Spearman correlational analyses.

^10^
In Stage 1 of the registered report, we wrote: “there are 21 focal tests (six correlations for Hypothesis 1, four correlations for Hypothesis 2, nine correlations for Hypothesis 3, and two correlations for Hypothesis 4.)”. However, when we analyzed the data in Stage 2, we found that the EEG markers used in Hypothesis 4 were the same as in Hypothesis 3. The difference was that, unlike Hypothesis 3, which was concerned with approach motivation, Hypothesis 4 was concerned with avoidance motivation. Therefore, there are only 19 focal tests in the article. We consider the influence of the change in the “Limitations” subsection of the Discussion section.
