# Supplementary material for: There is a party in my head and no one is invited: Resting‐state electrocortical activity and solitude
Source: J Pers. 2023 Aug 14;93(1):155–73. doi: 10.1111/jopy.12876 (PMC11705508; doi:10.1111/jopy.12876)
Supplement: Supplementary file 1 — Appendix S1 [file JOPY-93-155-s001.docx]

**Table 1**

*Spearman’s Correlation Between Self-Reported Approach-Avoidance Motivation Measures and Motivation-Related EEG Neurophysiological Markers*

|  | BAS | BIS | SAPM | SAIM |
| --- | --- | --- | --- | --- |
| FAA | .084 | .071 | -.086 | -.171^†^ |
| BP | .005 | .202^*^ | .141 | .079 |
| PFTA | .073 | -.022 | -.111 | -.083 |

*Note.* BAS: Behavioral Approach System; BIS: Behavioral Inhibition System; SAPM: Social Approach Motivation; SAIM: Social Avoidance Motivation; FAA: Frontal Alpha Asymmetry; BP: Beta Power; PFTA: Posterior versus Frontal EEG Theta Activity. ^*^*p* < .05, ^†^*p* < .01.

**Figure 1**

*Equivalence Tests for the Spearman’s Correlations*


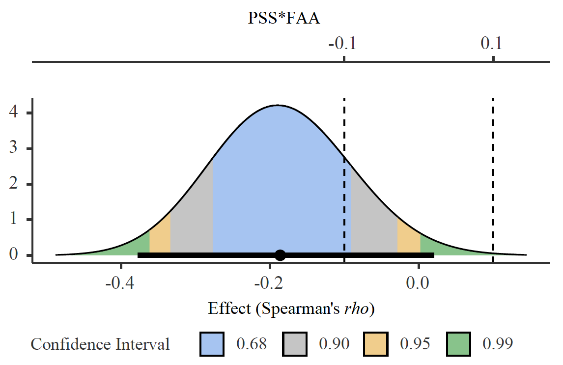

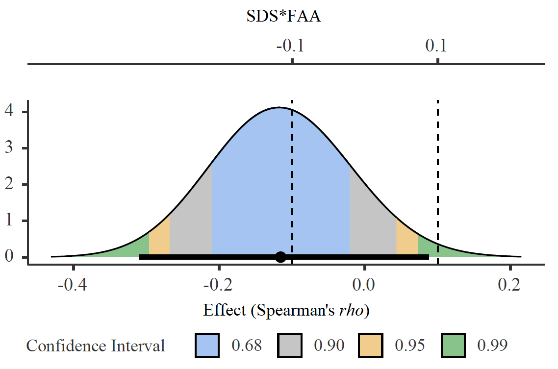

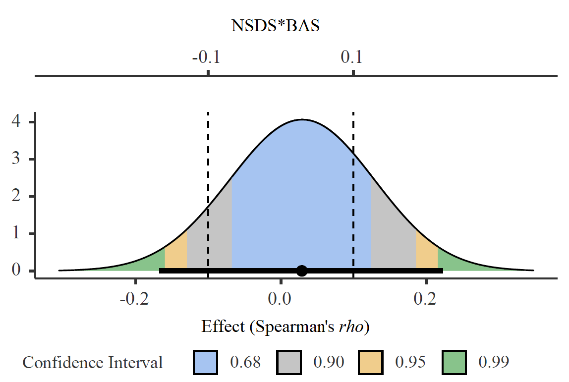

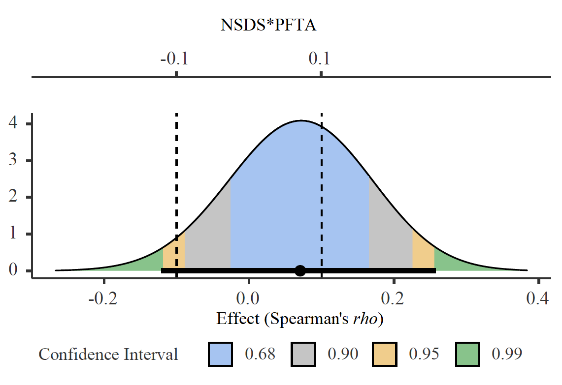

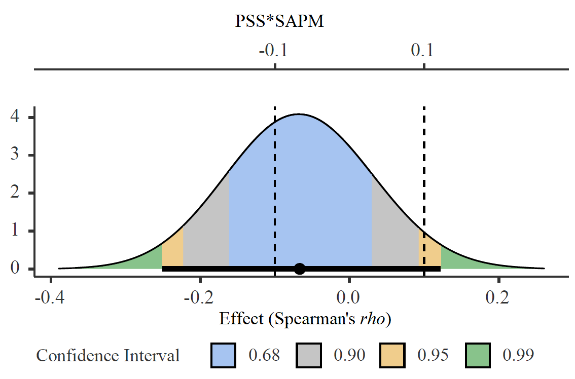

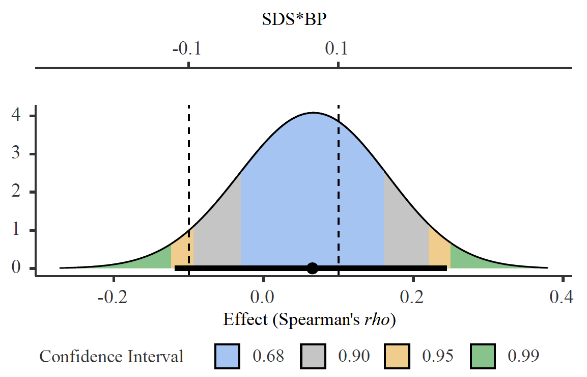

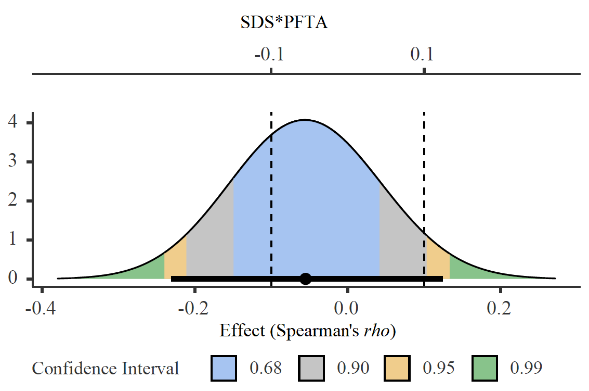

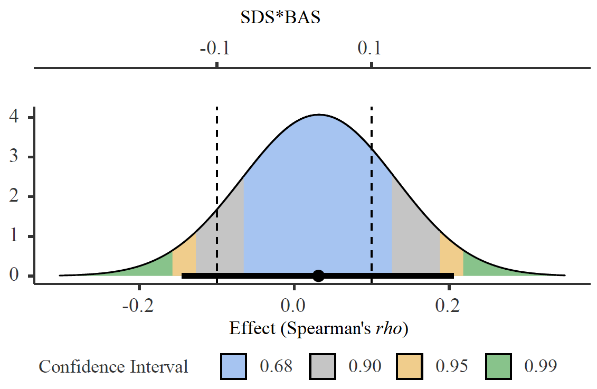

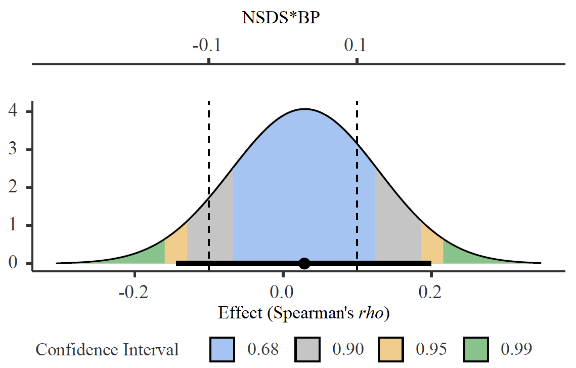

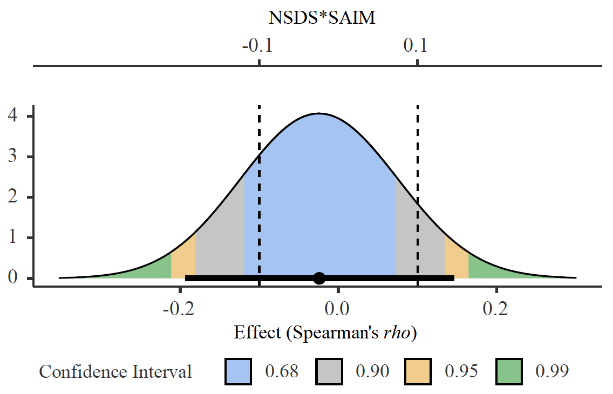

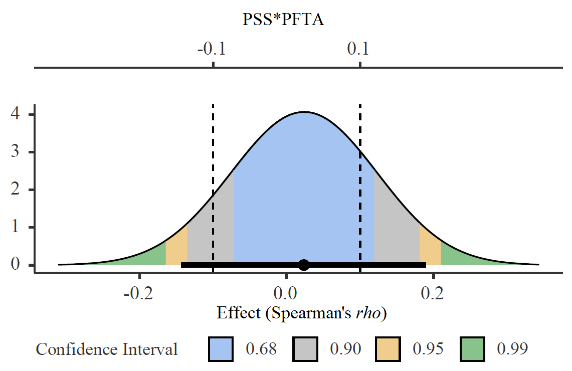

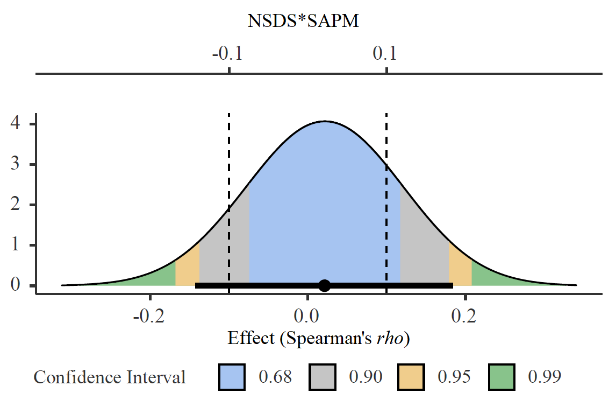

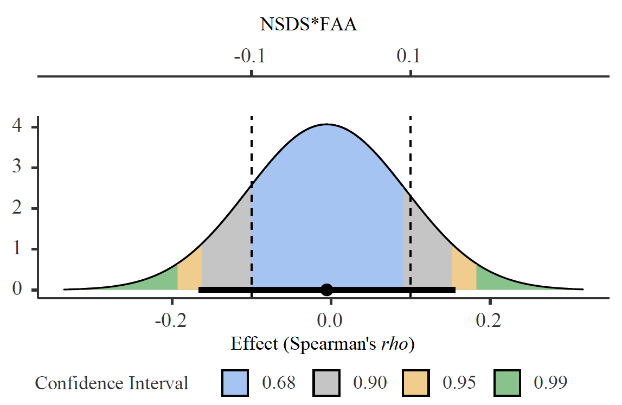

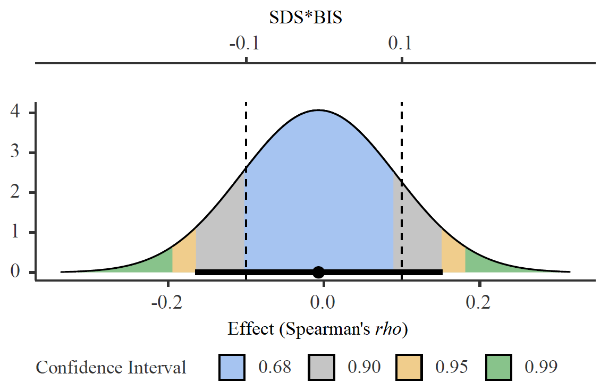


*Note:* PSS: Preference for Solitude Scale; SDS: Self-determined solitude Subscale; NSDS: Non-self-determined Solitude Subscale; BAS: Behavioural Approach System; BIS: Behavioural Inhibition System; SAPM: Social Approach Motivation; SAIM: Social Avoidance Motivation; FAA: Frontal Alpha Asymmetry; BP: Beta Power; PFTA: Posterior versus Frontal EEG Theta Activity. In the plots, the thick horizontal lines indicated the confidence intervals from the two one-sided tests (TOST) procedure, and the range of confidence intervals depends on the corrected *p* values (i.e., B-H critical values). Take SDS*FAA plot for example, the *Confidence Interval* was 96.4% since the B-H critical value was 0.018. The dashed vertical lines indicated the equivalence bounds.
